# Poly[tetra­kis[μ_2_-1,3-bis­(4-pyrid­yl)propane-κ^2^ *N*:*N*′]dichloridobis(phenyl­acetato)dimanganese(II)]

**DOI:** 10.1107/S1600536810003466

**Published:** 2010-02-03

**Authors:** Ji-Yong Liu, Wei Xu

**Affiliations:** a Center of Applied Solid State Chemistry Research, Ningbo University, Ningbo, Zhejiang 315211, People’s Republic of China

## Abstract

In the title compound, [Mn_2_(C_8_H_7_O_2_)_2_Cl_2_(C_13_H_14_N_2_)_4_]_*n*_, the two Mn^II^ atoms lie on inversion centers and are connected by the *N*-heterocyclic ligands into a wave-like lamellar framework structure. One Mn^II^ atom is covalently bonded to two Cl atoms and the other to two benzyl­acetate anions; both Mn atoms show distorted octahedral coordinations.

## Related literature

For general background to the use of poly-pyridyl ligand linkers such as 4,4′-bipyridine in the rational design and assembly of coordination polymers, see: Biradha *et al.* (2006 ▶). For related structures, see: Carlucci *et al.* (2002 ▶).
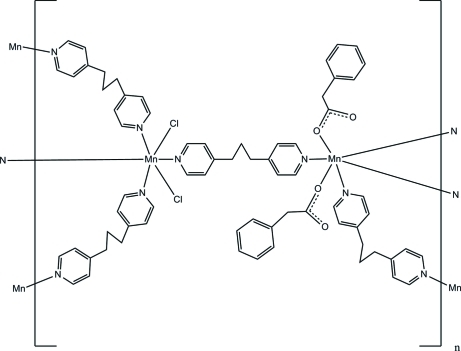


## Experimental

### 

#### Crystal data

[Mn_2_(C_8_H_7_O_2_)_2_Cl_2_(C_13_H_14_N_2_)_4_]
*M* *_r_* = 1244.10
Triclinic, 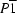

*a* = 9.5594 (5) Å
*b* = 13.0091 (6) Å
*c* = 13.8484 (6) Å
α = 69.202 (4)°
β = 86.318 (4)°
γ = 69.910 (5)°
*V* = 1508.74 (13) Å^3^
*Z* = 1
Mo *K*α radiation
μ = 0.56 mm^−1^
*T* = 293 K
0.48 × 0.46 × 0.23 mm

#### Data collection

Oxford Diffraction Xcalibur (Atlas Gemini ultra) diffractometer
Absorption correction: multi-scan (*CrysAlis RED*; Oxford Diffraction, 2009 ▶) *T*_min_ = 0.77, *T*_max_ = 0.88
10180 measured reflections
5303 independent reflections
4041 reflections with *I* > 2σ(*I*)
*R*_int_ = 0.021

#### Refinement

*R*[*F* ^2^ > 2σ(*F* ^2^)] = 0.030
*wR*(*F* ^2^) = 0.069
*S* = 0.96
5303 reflections
383 parameters
H-atom parameters constrained
Δρ_max_ = 0.23 e Å^−3^
Δρ_min_ = −0.19 e Å^−3^

### 

Data collection: *CrysAlis PRO* (Oxford Diffraction, 2009 ▶); cell refinement: *CrysAlis PRO*; data reduction: *CrysAlis PRO*; program(s) used to solve structure: *SHELXS97* (Sheldrick, 2008 ▶); program(s) used to refine structure: *SHELXL97* (Sheldrick, 2008 ▶); molecular graphics: *OLEX2* (Dolomanov *et al.*, 2009 ▶); software used to prepare material for publication: *OLEX2*.

## Supplementary Material

Crystal structure: contains datablocks global, I. DOI: 10.1107/S1600536810003466/ng2721sup1.cif

Structure factors: contains datablocks I. DOI: 10.1107/S1600536810003466/ng2721Isup2.hkl

Additional supplementary materials:  crystallographic information; 3D view; checkCIF report

## Figures and Tables

**Fig. 1.**
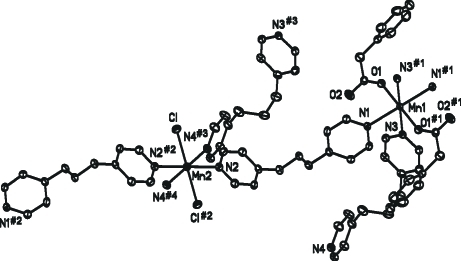
ORTEP view of the title compound. The dispalcement ellipsoids are drawn at 30% probability level [Symmetry codes: (#1) -x + 2, -y + 2, -z +1; (#2) -x + 1, -y + 1, -z; (#3) -x + 2, -y + 1, -z; (#4) x - 1, y, z; (#5) x + 1, y, z].

**Fig. 2.**
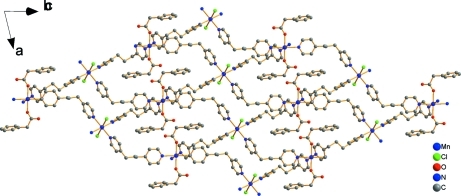
The two-dimensional layer of the compound. Hydrogen atoms are omitted for clarity.

## supplementary crystallographic information

### Comment

Over the past few decades, Some poly-pyridyl ligand linkers such as 4,4'-bipyridine have been extensively studied for rational design and assembly of coordination polymers [Biradha *et al.* (2006)]. However, few studies have been done to 1,3-bis(4-pyridyl)propane (bpp), which has analogous structures to 4,4'-bipyridine ligands. In this paper, we report the synthesis and crystal structure of the title compound.

The molecular unit consists of two Mn^2+^ ions (namely Mn1 and Mn2), four bpp molecules, two phenylacetate anions and two Cl^-^ anions. The Mn1 and Mn2 atoms both sit at symmetry inversion centers. Each Mn1 atom is coordinated by four N atoms from different bpp ligands and two two oxygen atoms of monodentate phenylacetate ligands to form a MnN_4_O_2_ chromophore with oxygen atoms occupied the axial positions. The coordination environment of Mn2 is completed by four N atoms and two Cl^-^ anions, forming a MnN_4_Cl_2_ chromophore, whose axial positions defined by Cl^-^ anions. The coordination environment of each Mn(II) could be best describes as distorted octahedral geometry, and the slight distortion is reflected on the cisoid angles [84.37 (5)-95.63 (5)°].

It's noting that the flexible bpp ligands presents two different conformations [Carlucci *et al.* (2002)], with rational N···N distance 9.223Å for TG bpp [torsion angles of 66.1 (4) and 174.1 (4)°] and 8.091Å for GG' bpp [torsion angles of 75.9 (5) and 163.7 (4)°], which lead to different distances of the adjacent Mn1 and Mn2 (13.065 and 10.930 Å). The Mn1 and Mn2 atoms are connected by bpp ligands into a wave-like lamellar framework structure in rectangle (4, 4) topology (Fig.2), which is further stacked into a 3D supramolecular architecture linked by the C—H···O and C—H···Cl hydrogen bonding interactions.

### Experimental

A mixture of MnCl_2_.4H_2_O (0.1977 g, 1.00 mmol) with phenylacetic acid (0.2731 g, 2.00 mmol), 1,3-bis(4-pyridyl)propane (0.1976 g, 1.00 mmol) and NaOH (0.0805 g, 2.00 mmol), in the molar ratio 1:2:1:2, and water (10 ml) was placed in a Parr Teflonlined stainless steel vessel (25 ml); the vessel was sealed and heated to 433 K for 3 d, and the reaction mixture was cooled to room temperature, yellow crystals were obtained from the filtrate after a few days.

### Refinement

H atoms bonded to C atoms were palced in geometrically calculated positionand were refined using a riding model, with *U*_iso_(H) = 1.2 Ueq(C).

### Crystal data

**Table:**

[Mn_2_(C_8_H_7_O_2_)_2_Cl_2_(C_13_H_14_N_2_)_4_]	*Z* = 1
*M**_r_* = 1244.10	*F*(000) = 650
Triclinic, *P*1	*D*_x_ = 1.369 Mg m^−^^3^
Hall symbol: -P 1	Mo *K*α radiation, λ = 0.71073 Å
*a* = 9.5594 (5) Å	Cell parameters from 5358 reflections
*b* = 13.0091 (6) Å	θ = 3.3–29.3°
*c* = 13.8484 (6) Å	µ = 0.56 mm^−^^1^
α = 69.202 (4)°	*T* = 293 K
β = 86.318 (4)°	Block, yellow
γ = 69.910 (5)°	0.48 × 0.46 × 0.23 mm
*V* = 1508.74 (13) Å^3^	

### Data collection

**Table:**

Oxford Diffraction Xcalibur (Atlas Gemini ultra) diffractometer	5303 independent reflections
Radiation source: fine-focus sealed tube	4041 reflections with *I* > 2σ(*I*)
graphite	*R*_int_ = 0.021
Detector resolution: 10.3592 pixels mm^-1^	θ_max_ = 25.0°, θ_min_ = 3.4°
ω scans	*h* = −11→11
Absorption correction: multi-scan (*CrysAlis RED*; Oxford Diffraction, 2009)	*k* = −13→15
*T*_min_ = 0.77, *T*_max_ = 0.88	*l* = −16→16
10180 measured reflections	

### Refinement

**Table:**

Refinement on *F*^2^	Secondary atom site location: difference Fourier map
Least-squares matrix: full	Hydrogen site location: inferred from neighbouring sites
*R*[*F*^2^ > 2σ(*F*^2^)] = 0.030	H-atom parameters constrained
*wR*(*F*^2^) = 0.069	*w* = 1/[σ^2^(*F*_o_^2^) + (0.0355*P*)^2^] where *P* = (*F*_o_^2^ + 2*F*_c_^2^)/3
*S* = 0.96	(Δ/σ)_max_ < 0.001
5303 reflections	Δρ_max_ = 0.23 e Å^−^^3^
383 parameters	Δρ_min_ = −0.19 e Å^−^^3^
0 restraints	Extinction correction: *SHELXL97* (Sheldrick, 2008), Fc^*^=kFc[1+0.001xFc^2^λ^3^/sin(2θ)]^-1/4^
Primary atom site location: structure-invariant direct methods	Extinction coefficient: 0.0064 (8)

### Special details

**Table:**

Geometry. All esds (except the esd in the dihedral angle between two l.s. planes) are estimated using the full covariance matrix. The cell esds are taken into account individually in the estimation of esds in distances, angles and torsion angles; correlations between esds in cell parameters are only used when they are defined by crystal symmetry. An approximate (isotropic) treatment of cell esds is used for estimating esds involving l.s. planes.
Refinement. Refinement of *F*^2^ against ALL reflections. The weighted *R*-factor wR and goodness of fit *S* are based on *F*^2^, conventional *R*-factors *R* are based on *F*, with *F* set to zero for negative *F*^2^. The threshold expression of *F*^2^ > σ(*F*^2^) is used only for calculating *R*-factors(gt) etc. and is not relevant to the choice of reflections for refinement. *R*-factors based on *F*^2^ are statistically about twice as large as those based on *F*, and *R*- factors based on ALL data will be even larger.

### Fractional atomic coordinates and isotropic or equivalent isotropic displacement parameters (Å^2^)

**Table:**

	*x*	*y*	*z*	*U*_iso_*/*U*_eq_	
Mn1	1.0000	1.0000	0.5000	0.02534 (11)	
Mn2	0.5000	0.5000	0.0000	0.02969 (12)	
Cl	0.34313 (5)	0.44105 (5)	0.14744 (4)	0.04018 (14)	
O1	0.75819 (12)	1.08965 (11)	0.46913 (9)	0.0362 (3)	
O2	0.64269 (15)	1.08883 (14)	0.33545 (10)	0.0522 (4)	
N1	1.00849 (15)	0.85804 (12)	0.42979 (11)	0.0288 (3)	
N2	0.69038 (16)	0.45962 (13)	0.12058 (11)	0.0312 (4)	
N3	1.05314 (16)	1.09985 (13)	0.33750 (11)	0.0299 (3)	
N4	1.40150 (16)	0.69381 (14)	−0.00685 (12)	0.0352 (4)	
C1	1.1512 (2)	0.71106 (18)	0.36191 (16)	0.0417 (5)	
H1	1.2449	0.6678	0.3479	0.050*	
C2	1.1387 (2)	0.79193 (18)	0.40728 (15)	0.0396 (5)	
H2	1.2256	0.8012	0.4232	0.047*	
C3	0.8877 (2)	0.84106 (17)	0.40593 (15)	0.0383 (5)	
H3	0.7952	0.8851	0.4207	0.046*	
C4	0.8926 (2)	0.76112 (18)	0.36018 (16)	0.0412 (5)	
H4	0.8043	0.7531	0.3450	0.049*	
C5	1.0260 (2)	0.69363 (16)	0.33697 (14)	0.0313 (4)	
C6	1.0345 (2)	0.60283 (17)	0.29095 (15)	0.0380 (5)	
H6B	1.1264	0.5864	0.2560	0.046*	
H6A	0.9517	0.6335	0.2398	0.046*	
C7	1.0295 (2)	0.49008 (16)	0.37315 (14)	0.0334 (4)	
H7A	0.9340	0.5064	0.4039	0.040*	
H7B	1.1066	0.4643	0.4273	0.040*	
C8	1.0510 (2)	0.38992 (16)	0.33360 (14)	0.0324 (4)	
H8B	1.1438	0.3762	0.2993	0.039*	
H8A	1.0597	0.3191	0.3923	0.039*	
C9	0.92566 (19)	0.41373 (15)	0.25951 (13)	0.0282 (4)	
C10	0.9306 (2)	0.46458 (17)	0.15395 (14)	0.0349 (5)	
H10	1.0132	0.4847	0.1270	0.042*	
C11	0.8136 (2)	0.48548 (17)	0.08854 (14)	0.0357 (5)	
H11	0.8204	0.5197	0.0178	0.043*	
C12	0.6865 (2)	0.40978 (17)	0.22292 (15)	0.0379 (5)	
H12	0.6029	0.3901	0.2480	0.045*	
C13	0.7992 (2)	0.38592 (17)	0.29348 (14)	0.0368 (5)	
H13	0.7904	0.3511	0.3639	0.044*	
C14	1.0025 (2)	1.17152 (17)	0.15429 (14)	0.0367 (5)	
H14	0.9354	1.1924	0.0991	0.044*	
C15	0.9625 (2)	1.13025 (16)	0.25450 (14)	0.0339 (4)	
H15	0.8686	1.1232	0.2649	0.041*	
C16	1.1850 (2)	1.11445 (17)	0.31863 (15)	0.0354 (5)	
H16	1.2483	1.0968	0.3749	0.043*	
C17	1.2331 (2)	1.15408 (17)	0.22097 (15)	0.0391 (5)	
H17	1.3266	1.1620	0.2126	0.047*	
C18	1.1416 (2)	1.18203 (16)	0.13546 (15)	0.0352 (5)	
C19	1.1965 (3)	1.21582 (18)	0.02808 (16)	0.0487 (6)	
H19B	1.2622	1.2595	0.0242	0.058*	
H19A	1.1122	1.2661	−0.0217	0.058*	
C20	1.2812 (2)	1.10655 (19)	0.00013 (17)	0.0507 (6)	
H20B	1.3367	1.1287	−0.0602	0.061*	
H20A	1.3523	1.0496	0.0570	0.061*	
C21	1.1775 (3)	1.0502 (2)	−0.02233 (18)	0.0550 (6)	
H21A	1.1016	1.0501	0.0277	0.066*	
H21B	1.1275	1.0973	−0.0905	0.066*	
C22	1.2571 (2)	0.92665 (19)	−0.01826 (16)	0.0416 (5)	
C23	1.3214 (2)	0.89998 (18)	−0.10331 (15)	0.0417 (5)	
H23	1.3170	0.9596	−0.1662	0.050*	
C24	1.3917 (2)	0.78443 (18)	−0.09375 (15)	0.0365 (5)	
H24	1.4348	0.7688	−0.1514	0.044*	
C25	1.3406 (2)	0.71987 (19)	0.07506 (15)	0.0462 (5)	
H25	1.3463	0.6586	0.1370	0.055*	
C26	1.2703 (2)	0.8323 (2)	0.07211 (17)	0.0516 (6)	
H26	1.2310	0.8454	0.1316	0.062*	
C27	0.64387 (19)	1.11445 (16)	0.41277 (14)	0.0309 (4)	
C28	0.4906 (2)	1.1779 (2)	0.44208 (17)	0.0562 (6)	
H28B	0.4395	1.2429	0.3804	0.067*	
H28A	0.4351	1.1245	0.4604	0.067*	
C29	0.47920 (19)	1.22539 (19)	0.52763 (16)	0.0391 (5)	
C30	0.5024 (2)	1.1515 (2)	0.62991 (18)	0.0489 (6)	
H30	0.5273	1.0713	0.6462	0.059*	
C31	0.4885 (2)	1.1967 (3)	0.70858 (19)	0.0643 (7)	
H31	0.5078	1.1462	0.7772	0.077*	
C32	0.4469 (3)	1.3141 (3)	0.6860 (3)	0.0713 (9)	
H32	0.4356	1.3440	0.7390	0.086*	
C33	0.4220 (3)	1.3873 (3)	0.5859 (3)	0.0703 (8)	
H33	0.3929	1.4677	0.5704	0.084*	
C34	0.4393 (2)	1.3440 (2)	0.50720 (19)	0.0544 (6)	
H34	0.4239	1.3955	0.4389	0.065*	

### Atomic displacement parameters (Å^2^)

**Table:**

	*U*^11^	*U*^22^	*U*^33^	*U*^12^	*U*^13^	*U*^23^
Mn1	0.0254 (2)	0.0287 (2)	0.0267 (2)	−0.00973 (17)	0.00331 (16)	−0.01524 (19)
Mn2	0.0266 (2)	0.0352 (2)	0.0289 (2)	−0.01084 (18)	0.00248 (17)	−0.0132 (2)
Cl	0.0320 (3)	0.0543 (3)	0.0350 (3)	−0.0170 (2)	0.0077 (2)	−0.0155 (3)
O1	0.0243 (6)	0.0468 (9)	0.0401 (8)	−0.0071 (6)	−0.0008 (6)	−0.0228 (7)
O2	0.0481 (8)	0.0678 (11)	0.0427 (9)	−0.0074 (8)	−0.0051 (7)	−0.0328 (9)
N1	0.0308 (8)	0.0281 (9)	0.0316 (9)	−0.0120 (7)	0.0024 (7)	−0.0137 (8)
N2	0.0319 (8)	0.0328 (9)	0.0306 (9)	−0.0118 (7)	0.0016 (7)	−0.0126 (8)
N3	0.0316 (8)	0.0299 (9)	0.0323 (9)	−0.0111 (7)	0.0041 (7)	−0.0157 (8)
N4	0.0369 (9)	0.0394 (10)	0.0313 (9)	−0.0116 (8)	0.0017 (7)	−0.0162 (9)
C1	0.0298 (10)	0.0485 (13)	0.0595 (14)	−0.0130 (10)	0.0121 (10)	−0.0356 (12)
C2	0.0279 (10)	0.0485 (13)	0.0573 (13)	−0.0175 (10)	0.0071 (9)	−0.0326 (12)
C3	0.0276 (10)	0.0374 (12)	0.0560 (13)	−0.0078 (9)	0.0054 (9)	−0.0272 (11)
C4	0.0290 (10)	0.0444 (13)	0.0621 (14)	−0.0134 (9)	−0.0010 (9)	−0.0313 (12)
C5	0.0374 (10)	0.0305 (11)	0.0317 (10)	−0.0139 (9)	0.0035 (8)	−0.0156 (9)
C6	0.0477 (12)	0.0397 (12)	0.0384 (12)	−0.0200 (10)	0.0090 (9)	−0.0237 (11)
C7	0.0343 (10)	0.0407 (12)	0.0330 (11)	−0.0143 (9)	0.0011 (8)	−0.0205 (10)
C8	0.0366 (10)	0.0281 (11)	0.0325 (10)	−0.0093 (9)	−0.0032 (8)	−0.0119 (9)
C9	0.0333 (10)	0.0217 (10)	0.0327 (11)	−0.0074 (8)	−0.0003 (8)	−0.0146 (9)
C10	0.0369 (11)	0.0421 (12)	0.0340 (11)	−0.0220 (10)	0.0049 (9)	−0.0153 (10)
C11	0.0419 (11)	0.0412 (12)	0.0268 (10)	−0.0196 (10)	0.0027 (9)	−0.0101 (10)
C12	0.0340 (11)	0.0458 (13)	0.0376 (12)	−0.0199 (10)	0.0063 (9)	−0.0138 (11)
C13	0.0422 (11)	0.0447 (13)	0.0263 (10)	−0.0192 (10)	0.0035 (9)	−0.0118 (10)
C14	0.0438 (12)	0.0343 (11)	0.0313 (11)	−0.0092 (9)	0.0007 (9)	−0.0144 (10)
C15	0.0292 (10)	0.0374 (12)	0.0375 (12)	−0.0110 (9)	0.0036 (9)	−0.0168 (10)
C16	0.0346 (10)	0.0369 (12)	0.0409 (12)	−0.0142 (9)	0.0028 (9)	−0.0191 (10)
C17	0.0376 (11)	0.0361 (12)	0.0499 (13)	−0.0185 (10)	0.0108 (10)	−0.0182 (11)
C18	0.0480 (12)	0.0213 (10)	0.0385 (11)	−0.0126 (9)	0.0128 (10)	−0.0141 (10)
C19	0.0703 (15)	0.0333 (12)	0.0449 (13)	−0.0235 (11)	0.0222 (11)	−0.0144 (11)
C20	0.0639 (14)	0.0472 (14)	0.0469 (13)	−0.0228 (12)	0.0251 (11)	−0.0234 (12)
C21	0.0581 (14)	0.0504 (15)	0.0564 (15)	−0.0063 (12)	0.0010 (11)	−0.0305 (13)
C22	0.0422 (11)	0.0436 (13)	0.0434 (13)	−0.0093 (10)	0.0005 (10)	−0.0253 (12)
C23	0.0496 (12)	0.0405 (13)	0.0356 (11)	−0.0136 (10)	0.0021 (9)	−0.0160 (11)
C24	0.0412 (11)	0.0404 (12)	0.0309 (11)	−0.0135 (10)	0.0042 (9)	−0.0170 (11)
C25	0.0618 (14)	0.0475 (14)	0.0290 (11)	−0.0181 (11)	0.0048 (10)	−0.0142 (11)
C26	0.0678 (15)	0.0540 (15)	0.0366 (13)	−0.0148 (12)	0.0114 (11)	−0.0276 (13)
C27	0.0320 (10)	0.0283 (11)	0.0331 (11)	−0.0114 (8)	0.0018 (9)	−0.0107 (9)
C28	0.0266 (11)	0.0852 (18)	0.0659 (15)	−0.0084 (11)	0.0010 (10)	−0.0474 (15)
C29	0.0187 (9)	0.0516 (14)	0.0517 (14)	−0.0080 (9)	0.0041 (9)	−0.0280 (12)
C30	0.0305 (11)	0.0498 (14)	0.0603 (15)	−0.0047 (10)	0.0035 (10)	−0.0213 (13)
C31	0.0351 (12)	0.100 (2)	0.0500 (14)	−0.0094 (14)	0.0028 (11)	−0.0306 (16)
C32	0.0425 (14)	0.112 (3)	0.094 (2)	−0.0270 (16)	0.0149 (14)	−0.078 (2)
C33	0.0529 (15)	0.0646 (19)	0.119 (3)	−0.0260 (14)	0.0207 (16)	−0.059 (2)
C34	0.0408 (12)	0.0527 (15)	0.0654 (16)	−0.0159 (11)	0.0111 (11)	−0.0176 (14)

### Geometric parameters (Å, °)

**Table:**

Mn1—O1	2.1925 (11)	C11—H11	0.9300
Mn1—O1^i^	2.1925 (11)	C12—C13	1.380 (2)
Mn1—N3^i^	2.2891 (15)	C12—H12	0.9300
Mn1—N3	2.2891 (15)	C13—H13	0.9300
Mn1—N1	2.3504 (12)	C14—C15	1.380 (3)
Mn1—N1^i^	2.3505 (12)	C14—C18	1.382 (3)
Mn2—N4^ii^	2.3374 (15)	C14—H14	0.9300
Mn2—N4^iii^	2.3374 (15)	C15—H15	0.9300
Mn2—N2	2.3425 (14)	C16—C17	1.376 (3)
Mn2—N2^iv^	2.3425 (14)	C16—H16	0.9300
Mn2—Cl^iv^	2.5081 (5)	C17—C18	1.381 (3)
Mn2—Cl	2.5081 (5)	C17—H17	0.9300
O1—C27	1.263 (2)	C18—C19	1.508 (3)
O2—C27	1.2309 (19)	C19—C20	1.545 (3)
N1—C3	1.330 (2)	C19—H19B	0.9700
N1—C2	1.338 (2)	C19—H19A	0.9700
N2—C12	1.337 (2)	C20—C21	1.522 (3)
N2—C11	1.340 (2)	C20—H20B	0.9700
N3—C16	1.334 (2)	C20—H20A	0.9700
N3—C15	1.341 (2)	C21—C22	1.506 (3)
N4—C24	1.333 (2)	C21—H21A	0.9700
N4—C25	1.339 (2)	C21—H21B	0.9700
N4—Mn2^v^	2.3374 (15)	C22—C26	1.382 (3)
C1—C2	1.374 (2)	C22—C23	1.391 (2)
C1—C5	1.378 (2)	C23—C24	1.380 (3)
C1—H1	0.9300	C23—H23	0.9300
C2—H2	0.9300	C24—H24	0.9300
C3—C4	1.384 (2)	C25—C26	1.371 (3)
C3—H3	0.9300	C25—H25	0.9300
C4—C5	1.371 (2)	C26—H26	0.9300
C4—H4	0.9300	C27—C28	1.524 (3)
C5—C6	1.507 (2)	C28—C29	1.501 (2)
C6—C7	1.518 (3)	C28—H28B	0.9700
C6—H6B	0.9700	C28—H28A	0.9700
C6—H6A	0.9700	C29—C30	1.381 (3)
C7—C8	1.532 (2)	C29—C34	1.381 (3)
C7—H7A	0.9700	C30—C31	1.391 (3)
C7—H7B	0.9700	C30—H30	0.9300
C8—C9	1.504 (2)	C31—C32	1.360 (4)
C8—H8B	0.9700	C31—H31	0.9300
C8—H8A	0.9700	C32—C33	1.354 (4)
C9—C10	1.379 (2)	C32—H32	0.9300
C9—C13	1.385 (2)	C33—C34	1.372 (3)
C10—C11	1.376 (2)	C33—H33	0.9300
C10—H10	0.9300	C34—H34	0.9300
			
O1—Mn1—O1^i^	180.00 (8)	N2—C11—H11	118.0
O1—Mn1—N3^i^	86.18 (5)	C10—C11—H11	118.0
O1^i^—Mn1—N3^i^	93.82 (5)	N2—C12—C13	123.72 (16)
O1—Mn1—N3	93.82 (5)	N2—C12—H12	118.1
O1^i^—Mn1—N3	86.18 (5)	C13—C12—H12	118.1
N3^i^—Mn1—N3	180.00 (7)	C12—C13—C9	120.04 (17)
O1—Mn1—N1	94.53 (4)	C12—C13—H13	120.0
O1^i^—Mn1—N1	85.47 (4)	C9—C13—H13	120.0
N3^i^—Mn1—N1	95.63 (5)	C15—C14—C18	120.39 (17)
N3—Mn1—N1	84.37 (5)	C15—C14—H14	119.8
O1—Mn1—N1^i^	85.47 (4)	C18—C14—H14	119.8
O1^i^—Mn1—N1^i^	94.53 (4)	N3—C15—C14	122.83 (16)
N3^i^—Mn1—N1^i^	84.37 (5)	N3—C15—H15	118.6
N3—Mn1—N1^i^	95.63 (5)	C14—C15—H15	118.6
N1—Mn1—N1^i^	179.999 (2)	N3—C16—C17	124.04 (17)
N4^ii^—Mn2—N4^iii^	180.0	N3—C16—H16	118.0
N4^ii^—Mn2—N2	89.48 (5)	C17—C16—H16	118.0
N4^iii^—Mn2—N2	90.52 (5)	C16—C17—C18	119.62 (16)
N4^ii^—Mn2—N2^iv^	90.52 (5)	C16—C17—H17	120.2
N4^iii^—Mn2—N2^iv^	89.48 (5)	C18—C17—H17	120.2
N2—Mn2—N2^iv^	180.0	C17—C18—C14	116.66 (17)
N4^ii^—Mn2—Cl^iv^	90.26 (4)	C17—C18—C19	120.68 (17)
N4^iii^—Mn2—Cl^iv^	89.74 (4)	C14—C18—C19	122.58 (18)
N2—Mn2—Cl^iv^	91.14 (4)	C18—C19—C20	111.16 (17)
N2^iv^—Mn2—Cl^iv^	88.86 (4)	C18—C19—H19B	109.4
N4^ii^—Mn2—Cl	89.74 (4)	C20—C19—H19B	109.4
N4^iii^—Mn2—Cl	90.26 (4)	C18—C19—H19A	109.4
N2—Mn2—Cl	88.86 (4)	C20—C19—H19A	109.4
N2^iv^—Mn2—Cl	91.14 (4)	H19B—C19—H19A	108.0
Cl^iv^—Mn2—Cl	180.0	C21—C20—C19	112.69 (18)
C27—O1—Mn1	146.73 (11)	C21—C20—H20B	109.1
C3—N1—C2	115.84 (14)	C19—C20—H20B	109.1
C3—N1—Mn1	123.51 (11)	C21—C20—H20A	109.1
C2—N1—Mn1	120.59 (10)	C19—C20—H20A	109.1
C12—N2—C11	115.74 (15)	H20B—C20—H20A	107.8
C12—N2—Mn2	124.04 (11)	C22—C21—C20	113.31 (18)
C11—N2—Mn2	120.22 (12)	C22—C21—H21A	108.9
C16—N3—C15	116.38 (15)	C20—C21—H21A	108.9
C16—N3—Mn1	121.77 (12)	C22—C21—H21B	108.9
C15—N3—Mn1	121.06 (11)	C20—C21—H21B	108.9
C24—N4—C25	116.24 (17)	H21A—C21—H21B	107.7
C24—N4—Mn2^v^	122.37 (11)	C26—C22—C23	116.10 (19)
C25—N4—Mn2^v^	121.18 (14)	C26—C22—C21	120.97 (17)
C2—C1—C5	120.49 (17)	C23—C22—C21	122.9 (2)
C2—C1—H1	119.8	C24—C23—C22	119.6 (2)
C5—C1—H1	119.8	C24—C23—H23	120.2
N1—C2—C1	123.68 (16)	C22—C23—H23	120.2
N1—C2—H2	118.2	N4—C24—C23	124.00 (17)
C1—C2—H2	118.2	N4—C24—H24	118.0
N1—C3—C4	123.36 (16)	C23—C24—H24	118.0
N1—C3—H3	118.3	N4—C25—C26	123.3 (2)
C4—C3—H3	118.3	N4—C25—H25	118.4
C5—C4—C3	120.74 (16)	C26—C25—H25	118.4
C5—C4—H4	119.6	C25—C26—C22	120.80 (18)
C3—C4—H4	119.6	C25—C26—H26	119.6
C4—C5—C1	115.90 (15)	C22—C26—H26	119.6
C4—C5—C6	121.64 (15)	O2—C27—O1	125.80 (17)
C1—C5—C6	122.42 (16)	O2—C27—C28	114.92 (16)
C5—C6—C7	111.65 (14)	O1—C27—C28	119.24 (15)
C5—C6—H6B	109.3	C29—C28—C27	119.64 (15)
C7—C6—H6B	109.3	C29—C28—H28B	107.4
C5—C6—H6A	109.3	C27—C28—H28B	107.4
C7—C6—H6A	109.3	C29—C28—H28A	107.4
H6B—C6—H6A	108.0	C27—C28—H28A	107.4
C6—C7—C8	114.66 (14)	H28B—C28—H28A	106.9
C6—C7—H7A	108.6	C30—C29—C34	117.88 (19)
C8—C7—H7A	108.6	C30—C29—C28	120.6 (2)
C6—C7—H7B	108.6	C34—C29—C28	121.4 (2)
C8—C7—H7B	108.6	C29—C30—C31	120.1 (2)
H7A—C7—H7B	107.6	C29—C30—H30	119.9
C9—C8—C7	113.34 (15)	C31—C30—H30	119.9
C9—C8—H8B	108.9	C32—C31—C30	120.6 (3)
C7—C8—H8B	108.9	C32—C31—H31	119.7
C9—C8—H8A	108.9	C30—C31—H31	119.7
C7—C8—H8A	108.9	C33—C32—C31	119.6 (2)
H8B—C8—H8A	107.7	C33—C32—H32	120.2
C10—C9—C13	116.48 (16)	C31—C32—H32	120.2
C10—C9—C8	121.62 (15)	C32—C33—C34	120.6 (2)
C13—C9—C8	121.89 (16)	C32—C33—H33	119.7
C11—C10—C9	120.01 (16)	C34—C33—H33	119.7
C11—C10—H10	120.0	C33—C34—C29	121.1 (2)
C9—C10—H10	120.0	C33—C34—H34	119.4
N2—C11—C10	124.01 (17)	C29—C34—H34	119.4

Symmetry codes: (i) −*x*+2, −*y*+2, −*z*+1; (ii) −*x*+2, −*y*+1, −*z*; (iii) *x*−1, *y*, *z*; (iv) −*x*+1, −*y*+1, −*z*; (v) *x*+1, *y*, *z*.
